# Skin cancer surgery under local anesthesia: clinicopathologic characteristics and age-stratified outcomes in 533 patients

**DOI:** 10.55730/1300-0144.6198

**Published:** 2026-02-04

**Authors:** Menekşe KASTAMONİ BAŞKAN, Süleyman ÇEÇEN, Selçuk AKIN, Şaduman BALABAN ADIM, Güzin Yeşim ÖZGENEL

**Affiliations:** 1 Department of Plastic, Reconstructive and Aesthetic Surgery, Faculty of Medicine, Uludag University, Bursa, Turkiye; 2 Department of Pathology, Faculty of Medicine, Uludag University, Bursa, Turkiye

**Keywords:** Skin neoplasms, local anesthesia, surgical margins, reconstructive surgical procedures, aged

## Abstract

**Background/aim:**

Skin cancer is the most common malignancy worldwide, particularly among elderly individuals. Many lesions are detected at an early stage because of their visibility and can be excised under local anesthesia, thereby avoiding the risks associated with general anesthesia or sedation. This study aimed to evaluate patient- and tumor-related characteristics in skin cancer surgery performed under local anesthesia, with particular emphasis on age-related outcomes.

**Materials and methods:**

This retrospective study included 533 patients with 613 histopathologically confirmed skin cancers excised under local anesthesia between 2019 and 2024. Demographic, histopathological, and surgical variables were recorded, including age, sex, tumor location, measured tumor area, histologic subtype, surgical margin status, reconstruction technique, and comorbid conditions. Patients were stratified into three age groups (<60, 60–80, and >80 years) for subgroup analyses. Statistical analyses were performed using the Mann–Whitney U test, Kruskal–Wallis test, chi-square test, and Spearman correlation analysis (SPSS version 26.0; IBM Corp., Armonk, NY, USA), with statistical significance set at p < 0.05.

**Results:**

The mean patient age was 68.5 years. Most lesions (86.1%) were located in the head and neck region. Basal cell carcinoma (65.4%) and squamous cell carcinoma (21.0%) were the predominant histopathological diagnoses. Primary closure (43.8%) and full-thickness skin grafting (25.0%) were the most frequently performed reconstruction methods. Larger tumor area was significantly associated with positive surgical margins (p < 0.01), and tumor type was significantly associated with both surgical margin status and reconstruction method (p < 0.0001). Age showed a statistically significant positive correlation with tumor area (p = 2.22 × 10^−7^) and with the presence of comorbidities (p < 0.001). Subgroup analysis demonstrated a higher proportion of squamous cell carcinoma and a larger mean tumor area in patients older than 80 years. No local anesthesia-related complications occurred.

**Conclusion:**

Skin cancer surgery performed under local anesthesia appears to be safe and effective across age groups, including elderly and comorbid patients. Tumor type and tumor area were significantly associated with surgical margin status and reconstruction method. These findings support age-conscious and anesthesia-sparing approaches in oncologic dermatologic surgery.

## 1. Introduction

Skin cancers constitute the most common group of malignant tumors worldwide and represent a significant clinical burden, particularly in aging populations [1]. Fair skin type, advanced age, cumulative sun exposure, and genetic predisposition are well-established risk factors for the development of skin cancer [2]. Because changes in existing moles, ulceration, bleeding, or newly emerging lesions are often readily recognized by patients, many skin cancers are diagnosed at an early stage and can be managed with surgical excision under local anesthesia.

With the growing elderly population and increased lifetime ultraviolet exposure, the incidence of skin cancer continues to rise, particularly among older adults [3,4]. In this population, the use of local anesthesia offers distinct advantages by minimizing systemic risks associated with general anesthesia or sedation and by allowing procedures to be performed safely in an outpatient setting [5].

This study aimed to evaluate patient- and tumor-related characteristics in skin cancer surgery performed under local anesthesia, with particular emphasis on tumor area, histologic type, surgical margin status, and reconstruction methods. In addition, age-related patterns were analyzed to better characterize the association between aging, tumor characteristics, and surgical planning.

## 2. Materials and methods

This retrospective, single-center study included a total of 613 histopathologically confirmed skin cancer lesions excised under local anesthesia from 533 patients between 2019 and 2024. The study was approved by the Institutional Ethics Committee (approval no. 2025/4–29) and was conducted in accordance with the Declaration of Helsinki and the STROBE guidelines. All data were retrieved from electronic hospital records. No personally identifiable information was collected, and written informed consent was obtained at the time of treatment for both the surgical procedure and the use of anonymized data.

Malignant lesions removed under general anesthesia or sedation were excluded from the study. Benign and premalignant lesions accompanying malignant lesions excised under local anesthesia were excluded from the analysis. The analysis focused exclusively on the initial (primary) surgical procedures performed under local anesthesia. In cases with positive surgical margins, subsequent management decisions—such as reexcision or clinical follow-up—were made on an individual basis; however, these secondary interventions were not included in the present analysis. Patients were evaluated with respect to age, sex, lesion location, tumor area, histologic type, surgical margin status of the primary excision, reconstruction method, and comorbid conditions.

Local anesthesia was administered by the operating surgeon. For small lesions, a lidocaine-based local anesthetic solution was used. For larger defects requiring split-thickness skin grafting (STSG) or extensive flap reconstruction, diluted prilocaine-based infiltration was used in accordance with the principles of diluted (tumescent) local anesthesia described in the dermatologic surgery literature [5,6]. In these cases, one vial of prilocaine 2% (20 mL; 20 mg/mL, total dose 400 mg) was diluted in 100 mL of normal saline and infiltrated into the surgical field. The total administered dose was maintained within accepted safety limits, and patients were clinically monitored throughout the procedure. Supplemental lidocaine was administered when required to ensure adequate analgesia.

Statistical analyses were performed using SPSS version 26.0 (IBM Corp., Armonk, NY, USA). Quantitative variables were expressed as mean ± standard deviation, and categorical variables as frequencies and percentages. The Mann–Whitney U test and Kruskal–Wallis test were used to compare continuous variables, whereas the chi-square test was used to analyze categorical data. Correlations between continuous parameters were evaluated using Spearman’s correlation test. A p-value of <0.05 was considered statistically significant.

## 3. Results

**Figure 1:**
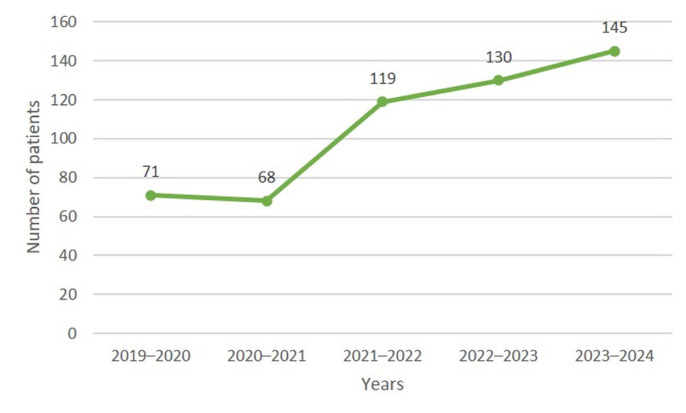
Annual distribution of patients undergoing skin cancer surgery under local anesthesia (2019–2024).

Of the 533 patients included in the study, 327 were male and 206 were female. The mean age was 68.53 years (range: 14–98 years). A single lesion was present in 483 patients, while 50 patients had multiple lesions. Among patients with multiple lesions, 34 had two lesions, eight had three lesions, four had four lesions, two had five lesions, and two had six lesions. A total of 613 skin cancer lesions were excised from 533 patients. The annual distribution of patients undergoing surgery is presented in Figure 1. A temporary decline was observed in 2020–2021, which coincided with the COVID-19 pandemic period. In recent years, an increase in patient numbers has been observed.

### 3.1. Lesion characteristics

The mean tumor area was 2.29 cm^2^ (range: 0.01–48.72 cm^2^). Of all lesions, 86.1% were located in the head and neck region, which are chronically sun-exposed areas. Detailed anatomical distribution is provided in Table 1. Histopathological evaluation identified basal cell carcinoma (BCC) as the most common histologic subtype (n = 401, 65.4%), followed by squamous cell carcinoma (SCC) (n = 129, 21.0%) and malignant melanoma (n = 33, 5.4%). Less frequent malignancies included basosquamous carcinoma (BSC), myxofibrosarcoma, mucinous adenocarcinoma, pleomorphic dermal sarcoma, and other rare tumor types (Table 2).

### 3.2. Reconstruction methods and surgical margin status

**Figure 2:**
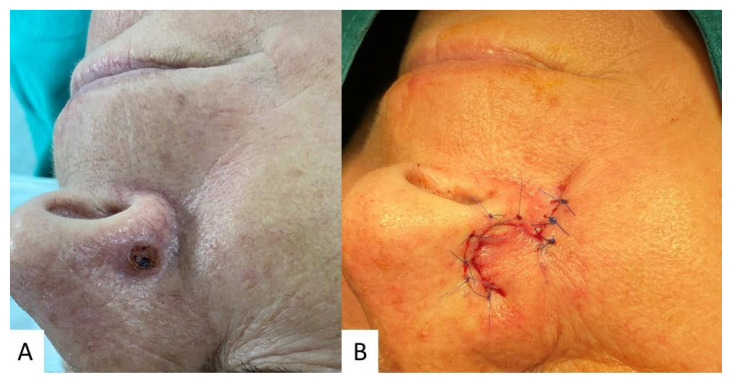
Representative case of flap reconstruction following skin cancer excision under local anesthesia. (A) Preoperative view of a basal cell carcinoma located in the right nasal alar region in a 66-year old female patient. (B) Postoperative view after tumor excision and reconstruction with a nasolabial flap.

**Figure 3:**
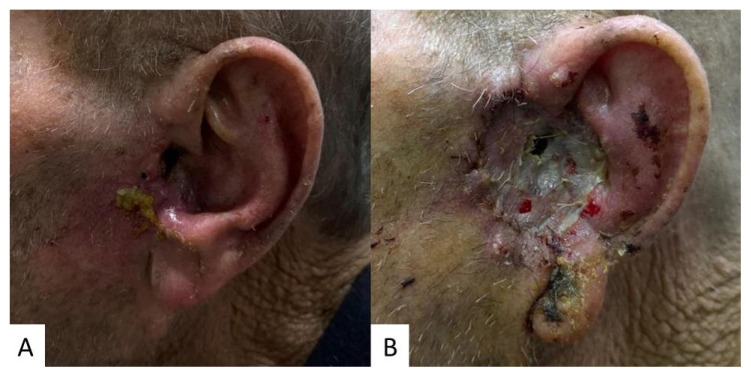
Representative case of reconstruction with a full-thickness skin graft following skin cancer excision under local anesthesia. (A) Preoperative view of a squamous cell carcinoma located in the left preauricular region in a 79-year old male patient. (B) Postoperative view after wide excision and reconstruction with a full-thickness skin graft.

Reconstruction methods varied according to defect size and anatomical location following tumor excision (Table 3). Primary closure was the most common method (n = 269, 43.8%), followed by full-thickness skin grafting (FTSG) (n = 153, 25.0%). Flap reconstruction was performed in 113 lesions (18.4%), including Limberg (rhomboid) flaps (n = 42), bilobed flaps (n = 20), fasciocutaneous advancement flaps (n = 19), nasolabial flaps (n = 17), and other regional flap techniques. A representative example of flap reconstruction following tumor excision under local anesthesia is shown in Figures 2A and 2B. An illustrative example of reconstruction using a full-thickness skin graft is presented in Figures 3A and 3B. Split-thickness skin grafts (STSG) were performed in 37 cases, wedge resections were performed in 26 cases, and healing by secondary intention was allowed in 15 defects. Surgical margins were histologically negative in 527 lesions (86.0%), while positive margins were detected in 86 (14.0%).

### 3.3. Comorbidities and local anesthesia safety

Comorbid conditions were frequently observed, with hypertension identified in 225 patients (42.2%) and diabetes mellitus in 95 patients (17.8%). No complications related to local anesthesia were observed in this cohort, which supports the safety of this approach in elderly and medically compromised patients within the context of the present study.

### 3.4. Age-related findings

Patients were stratified into three age groups: <60 years (n = 123), 60–80 years (n = 311), and > 80 years (n = 99). Age-related demographic and tumor characteristics are summarized in Table 4. Patients older than 80 years had a higher proportion of SCC (38.6%), a larger mean tumor area (3.42 cm^2^), and a greater proportion of lesions documented as recurrent at presentation (26%) compared with younger age groups (approximately 10% in each group). Despite the larger tumor area and higher proportion of SCC observed in advanced age, all procedures were completed under local anesthesia without major complications.

### 3.5. Statistical findings

The associations between clinicopathological parameters, surgical margins, reconstruction methods, and age are summarized in Table 5. Tumor area was significantly larger in lesions with positive surgical margins compared with those with negative margins (p < 0.01). Surgical margin status was significantly associated with tumor type (p < 0.0001) and reconstruction method (p < 0.0001). In addition, a significant association was observed between tumor type and reconstruction method (p < 0.0001). Tumor area differed significantly among reconstruction methods (p = 2.19 × 10−^13^). Patients with comorbid conditions were significantly older than those without comorbid conditions (p < 0.001). Furthermore, age showed a weak but statistically significant positive correlation with tumor area (p = 2.22 × 10−^7^).

## 4. Discussion

Skin cancers are among the most common malignancies in both women and men [7]. Because cutaneous changes are often readily recognized by patients and those around them, many skin cancers are diagnosed at an early stage; lesions detected early are frequently small enough to be excised under local anesthesia. In this study, consistent with previous reports, 65.4% of the lesions were identified as BCC and 21.0% as SCC, which is consistent with the predominance of nonmelanoma skin cancers reported in clinical practice [8].

Prolonged exposure to ultraviolet (UV) radiation is one of the most important risk factors for skin cancer. This is consistent with the higher frequency of lesion localization in the head and neck region. In our series, 86.1% of the lesions were located in this region. Advanced age is another major risk factor; in the present study, the mean patient age was 68.53 years, which is consistent with the World Health Organization definition of older age. Fair skin type and genetic predisposition also remain well-recognized contributors [9].

In elderly patients, the presence of systemic comorbid conditions such as diabetes and cardiovascular disease may influence anesthesia selection during surgical planning. Local anesthesia represents an appropriate option to minimize the potential risks associated with general anesthesia or sedation in this patient group [5]. It has also been reported to be safely used in pregnant patients for similar indications [10]. The sufficient duration of action, minimal systemic and local toxicity, and ability to provide postoperative analgesia have been reported to support the reliability of local anesthesia [5]. In the present study, no anesthesia-related complications were observed, which supports the safety of this approach within the context of the study cohort.

Although various local anesthetic agents and combinations have been described for diluted or tumescent infiltration techniques in the literature—most commonly lidocaine-based solutions—prilocaine has also been reported as a suitable alternative, particularly in European dermatologic surgery practice [5,6]. Rather than introducing a novel anesthetic technique, our aim was to provide methodological transparency and real-world clinical data regarding a simplified, prilocaine-based diluted local anesthesia approach in a large patient cohort. In this cohort, diluted prilocaine infiltration alone was sufficient to provide adequate analgesia for the majority of cases requiring wide excision and complex reconstruction under local anesthesia. Notably, no anesthesia-related adverse events were observed, even among elderly and comorbid patients. These findings suggest the feasibility and safety of a prilocaine-based diluted local anesthesia strategy when applied within accepted dose limits and appropriate clinical monitoring and are consistent with previous reports on the safety of tumescent local anesthesia in high-risk patient populations [5,11].

In addition to its anesthetic benefits, local anesthesia—particularly when lidocaine–adrenaline solutions are used—provides a hemostatic effect. Vasoconstriction induced by adrenaline has been shown to reduce intraoperative bleeding and operative time, which may be particularly beneficial in patients receiving anticoagulant therapy [5,12]. This may be particularly advantageous in elderly patients receiving anticoagulant therapy. Shorter surgical duration may reduce the risk of infection, and same-day discharge may help minimize nosocomial infections and associated healthcare costs [5,10]. Factors such as minimal equipment requirements, ease of application, and low cost further support the role of local anesthesia in clinical practice [13]. Moreover, reductions in hospital stay and overall economic burden have been associated with improved patient satisfaction [14].

Under local anesthesia, patients remain awake and cooperative throughout the procedure. Although patient cooperation may facilitate intraoperative flap planning, remaining awake may increase stress levels, which represents a potential limitation of local anesthesia [15]. In addition, local anesthesia is not suitable for infants, young children, mentally disabled patients, or uncooperative patients. The presence of a suppurative infection at the injection site also represents a limitation for the use of local anesthesia [10].

In our statistical analysis, several clinically meaningful associations were identified. Tumor area was significantly larger in lesions with positive surgical margins (p < 0.01), indicating an association between larger tumor area and margin positivity. Additionally, surgical margin status was significantly associated with tumor type (p < 0.0001), with BCC cases demonstrating higher proportions of negative margins compared with SCC and BSC. Reconstruction method was also significantly associated with tumor type (p < 0.0001); primary closure was more frequently performed in BCC cases, whereas grafts and flaps were more commonly used in tumors with more aggressive histologic features. Furthermore, tumor area differed significantly among reconstruction techniques (p = 2.19 × 10−^13^), with the largest mean tumor areas observed in lesions reconstructed by healing by secondary intention or grafting. A significant association was observed between comorbidity status and age, as patients with comorbid conditions were significantly older (p < 0.001). Lastly, although the correlation was weak, age was positively and significantly associated with tumor area (p = 2.22 × 10−^7^). These findings underscore the importance of tailoring surgical and anesthetic approaches according to tumor characteristics, patient age, and overall health status.

The age-stratified evaluation demonstrated that tumor area and the proportion of SCC increased with advancing age, which is consistent with literature emphasizing cumulative UV exposure and delayed diagnosis in elderly populations. Despite these more aggressive patterns, all procedures were completed under local anesthesia without major complications, which supports the safety and feasibility of this approach across age groups within the context of this study.

In addition to the descriptive findings, these results may have practical implications for surgical planning in elderly patients with skin cancer. The higher proportion of SCC and larger tumor areas observed in older age groups suggest that wider excisions and more complex reconstruction methods may be required in this population. These considerations are particularly relevant in elderly and comorbid patients, in whom balancing oncologic safety with functional preservation and anesthetic risk is essential. These findings support the importance of individualized surgical planning under local anesthesia, especially in older patients with SCC, in whom careful margin assessment and reconstruction strategies should be considered preoperatively.

Previous studies have consistently demonstrated the safety and high patient satisfaction associated with skin cancer surgery performed under local anesthesia. McKechnie reported 98% satisfaction in patients treated in a “see-and-treat” clinic model, supporting the practicality of local anesthesia in outpatient settings [16]. Similarly, Schnabl and colleagues evaluated over 700 elderly or multimorbid patients and reported that tumescent local anesthesia was associated with safe and well-tolerated outcomes, with systemic effects described as mild and primarily linked to preexisting comorbidities [5,11]. In parallel, Bordianu and Bobirca reported that the advantages of local anesthesia—such as reduced operative risk and faster recovery—outweighed its limitations [10]. Furthermore, patient preference studies by Neal et al. reported that the majority of individuals favored local anesthesia over general anesthesia, primarily due to lower cost and reduced perceived complication risk [17]. Alam et al. also reported that lidocaine doses used during skin cancer excision remained within accepted systemic safety limits, supporting the reliability of this approach [18]. Collectively, these findings align with the present results, suggesting that local anesthesia represents a safe, patient-centered, and efficient option for the surgical management of skin cancer across age groups.

Skin cancer surgery performed under local anesthesia appears to be a safe, effective, and cost-efficient treatment modality, including for elderly or comorbid patients within the context of this study. In this large retrospective cohort, tumor type and tumor area were significantly associated with reconstruction strategy and surgical margin status. The observed correlations between age, comorbidity status, and tumor characteristics underscore the importance of individualized treatment planning.

Nevertheless, the retrospective, single-center design limits generalizability. In addition, the analysis was limited to primary surgical excisions performed under local anesthesia. Although cases with positive margins underwent individualized subsequent management, including reexcision or clinical follow-up when indicated, these secondary interventions were not included in the analysis. Therefore, long-term oncologic outcomes related to margin status could not be evaluated within the scope of this study. Furthermore, multivariate predictive analyses were not performed, as the primary aim of this study was to explore descriptive associations between tumor characteristics, patient factors, and surgical outcomes in a real-world cohort. Accordingly, independent predictors of positive surgical margins could not be identified. Future multicenter, prospective studies with larger and more homogeneous subgroups and longer follow-up periods are warranted to validate these findings, assess long-term oncologic outcomes, and further evaluate patient-reported satisfaction with local anesthesia–based treatment.

These findings suggest the practicality of local anesthesia in oncologic dermatologic surgery and indicate a potential role in reducing perioperative risk and healthcare burden. The study contributes one of the largest single-center datasets analyzing awake skin cancer surgery and provides real-world evidence supporting anesthesia-sparing surgical approaches within the limitations of its retrospective design.

## Figures and Tables

**Table 1:** Anatomical distribution of lesion sites.

Anatomical sites of lesions	Number of lesions *(n = 613)*

Nose *(n = 144)*	
Dorsum	103
Ala nasi	30
Lateral	10
Tip	1

Ear *(n = 75)*	
Helix	38
Preauricular	21
Postauricular	10
Inferior	5
Lobe	1

Periorbital *(n = 62)*	
Inferior	28
Medial canthus	11
Lateral	11
Lower lid	10
Upper lid	2

Temporal region	41

Forehead	39

Cheek	39

Malar region	34

Scalp	29

Lip *(n = 29)*	
Lower lip	20
Upper lip	7
Lateral	2

Dorsal region	22

Upper extremity *(n = 21)*	
Forearm	8
Dorsum of the hand	5
Finger	2
Arm	2
Elbow	2
Shoulder	2

Lower extremity *(n = 21)*	
Thigh	7
Leg	7
Foot	4
Knee	3

Chin	17

Neck	9

Eyebrow	8

Chest	8

Lumbar region	4

Trunk	2

Inguinal region	2

Sacral region	2

Abdominal region	2

Axilla	1

Glabella	1

Nape of the neck	1

**Table 2:** Histopathological diagnosis.

Histological diagnosis	Number of lesions *(n = 613)*
Basal cell carcinoma	401
Squamous cell carcinoma	129
Malignant melanoma	33
Basosquamous carcinoma	22
Recurrent myxofibrosarcoma	8
Mucinous adenocarcinoma	5
Pleomorphic dermal sarcoma	5
Malignant proliferating trichilemmal tumor	4
Verrucous carcinoma	2
Metastatic tumor infiltration	2
Sebaceous carcinoma	1
Kaposi sarcoma	1

**Table 3:** Reconstruction types.

Reconstruction type	Number of lesions *(n = 613)*

Primary closure	269

Full thickness skin graft	153

Flap reconstruction *(n = 113)*	
Limberg (rhomboid) flap	42
Bilobed flap	20
Fasciocutaneous advancement flap	19
Nasolabial flap	17
V-Y advancement flap	4
Rotation flap	4
Other regional flap techniques	7

Split thickness skin graft	37

Wedge excision	26

Healing by secondary intention	15

**Table 4:** Demographic and tumor characteristics stratified by age group.

Parameter	<60 years (n = 123, 129 lesions)	60–80 years (n = 311, 357 lesions)	>80 years (n = 99, 127 lesions)
**Sex (M / F)**	66 / 57	196 / 115	65 / 34
**Tumor type**	BCC:83 (64.3%) SCC:20 (15.5%) Other:26 (20.1%)	BCC:256 (71.7%) SCC:59 (16.5%) Other:42 (11.7%)	BCC:62 (48.8%) SCC:49 (38.5%) Other:16 (12.5%)
**Lesion status**	Intact:115 (89.1%) Recurrent:14 (10.9%)	Intact:318 (89%) Recurrent:39 (11%)	Intact:94 (74%) Recurrent:33 (26%)
**Mean tumor area (cm** **^2^** **)**	2.06 (0.03–32)	1.85 (0.01–48.72)	3.42 (0.02–33)

M: male; F: female.

**Table 5:** Summary of statistical analyses.

Parameters	Statistical tests	p-value
**Tumor area and surgical margin status**	Mann-Whitney U test	<0.01
**Tumor type and surgical margin status**	Chi-square test	<0.0001
**Tumor type and reconstruction type**	Chi-square test	<0.0001
**Surgical margin status and reconstruction type**	Chi-square test	<0.0001
**Reconstruction type and tumor area**	Kruskal-Wallis	2.19 × 10^−13^
**Comorbidity status and age**	Mann-Whitney U	4.52 × 10^−6^
**Age and tumor area**	Spearman correlation analysis	2.22 × 10^−7^
